# Physical activity and colon cancer prevention: a meta-analysis

**DOI:** 10.1038/sj.bjc.6604917

**Published:** 2009-02-10

**Authors:** K Y Wolin, Y Yan, G A Colditz, I-M Lee

**Affiliations:** 1 Department of Surgery, Washington University School of Medicine, St Louis, MO, USA; 2 Division of Preventive Medicine, Brigham and Women's Hospital and Harvard Medical School, Boston, MA, USA; 3 Department of Epidemiology, Harvard School of Public Health, Boston, MA, USA

**Keywords:** physical activity, colon cancer, meta-analysis

## Abstract

Although an inverse association between physical activity and risk of colon cancer is well established, a formal estimate of the magnitude of this risk reduction that includes recent studies is not available. This analysis examines the association by sex and study design, restricting analyses to studies where data for colon cancer alone were available. The authors reviewed published studies through June 2008 examining the association between physical activity and risk of colon cancer. Heterogeneity and publication bias were evaluated and random effects models used to estimate relative risks (RR). Differences by sex and study design were evaluated. A total of 52 studies were included. An inverse association between physical activity and colon cancer was found with an overall relative risk (RR) of 0.76 (95% confidence interval (CI): 0.72, 0.81). For men, the RR was 0.76 (95% CI: 0.71, 0.82); for women, this was little different, (RR=0.79, 95% CI: 0.71, 0.88). The findings from case–control studies were stronger (RR=0.69, 95% CI: 0.65, 0.74) than for cohort studies (RR=0.83, 95% CI: 0.78, 0.88). This study confirms previous studies reporting an inverse association between physical activity and colon cancer in both men and women, and provides quantitative estimates of the inverse association.

Ample and consistent evidence exists for a significant, inverse association between physical activity and risk of developing colon cancer (International Agency for Research on Cancer WHO, 2002). Moreover, this association is plausibly supported by several biological mechanisms, including decreased inflammation, reduced intestinal transit time, decreased insulin-like growth factor levels, reduced hyperinsulinemia and modulated immune function with physical activity. Although several qualitative reviews of this literature have been conducted, an up-to-date meta-analysis of the studies of physical activity and colon cancer is not available (International Agency for Research on Cancer WHO, 2002; Slattery, 2004). One recent study quantitatively evaluated data on the relation between physical activity and risk of colon cancer, but also included colorectal cancer end points (Samad *et al*, 2005). No association has been consistently found for physical activity and rectal cancer, and the consensus is that one is unlikely to exist (International Agency for Research on Cancer WHO, 2002). Estimation of the risk reduction associated with physical activity separately for colon cancer is important for public health because the disease burden attributable to physical inactivity is likely underestimated by including rectal cancer end points. The World Health Organization conducted a meta-analysis using data from studies prior to 2000, and estimated that 16% of the global colon cancer disease burden is due to physical inactivity (Bull *et al*, 2004). However, this meta-analysis was conducted several years ago, and also did not investigate differences by study design. Thus, we conducted a meta-analysis to estimate the summary relative risk of colon cancer associated with physical activity, based on available studies to date.

## Materials and methods

A search was conducted on PubMed for all publications in English through June 2008. The search terms physical activity, exercise, and colon cancer were used. In addition, previous reviews of the data (Slattery, 2004; Samad *et al*, 2005; Lee and Oguma, 2006) and the reference lists of included studies were reviewed.

We included only case–control or cohort studies with a colon cancer end point. Studies that reported the findings for rectal cancer or for colon and rectal cancers combined (colorectal cancer) were excluded for the reason detailed above. We did not limit studies by type of physical activity; thus, studies measuring total physical activity, recreational or leisure-time physical activity, physical activity in commuting and occupational physical activity could all be included.

From each article, the study design, sample size, age range, years of follow-up or type of control sample (depending on study design), type of physical activity and results were abstracted. We also abstracted the variables that each study used in its most adjusted analysis. We extracted information on study quality, including whether the study evaluated the accuracy of the physical activity instrument, whether physical activity was quantified, whether medical records were used to confirm the outcome or was a death certificate or tumour registry used to identify the outcome, and loss to follow-up or study response rate. Data extraction was performed by a single investigator (KYW).

An indicator of study quality was created where one point was assigned to studies for evaluation of physical activity instrument accuracy (i.e., instrument has been assessed for reliability or validity), and one point for quantification of physical activity (measured levels including that based on self report *vs* qualitative description such as active *vs* inactive). All studies that reported on instrument accuracy found the instrument had fair reliability or validity. Studies that used medical records to evaluate the outcome received two points, whereas those that used a death certificate or tumour registry received one point. Studies that adjusted for age and some component of diet received one point; those that also adjusted for use of non-steroidal anti-inflammatory drugs or multivitamin use received an additional point. For cohort studies, those with loss to follow-up of less than 20% received one point. For case–control studies, those with a response rate over 70% for cases received 0.5 points, whereas those with a response rate of over 70% for controls received 0.5 points, for a total of 1 point. Thus, the maximum quality score was seven for both cohort and case–control studies. This indicator was based on one previously used by Oguma and Shinoda-Tagawa (2004).

### Data analysis

Random effects meta-analysis was used to allow for the heterogeneity of results across studies. Data were processed in SAS and the analyses were performed using R-package ‘meta’. We evaluated case–control and cohort studies separately, and also considered results for men and women separately when available. To evaluate the potential effects that increased screening might have, we conducted an exploratory analysis by time period. Finally, given the increasing interest in the effects of type of physical activity (Physical Activity Guidelines Advisory Committee, 2008), we conducted exploratory analysis separately for occupational and leisure-time physical activity for those studies where such data was available. In the main analyses, we did not adjust for study quality; in additional analyses to examine the impact of study quality, summary estimates across studies were adjusted for quality score. When multiple types of physical activity were reported we used a summary measure of total physical activity if provided in our main analysis. If multiple domain-specific physical activity results were reported, we included only the leisure-time physical activity in our primary analyses as this measure was available for most studies. When physical activity at different ages was recalled and assessed at a single time point, we used the age range that best represented adulthood (30–50 years old). As most studies reported relative risks (RR) or odds ratios (OR) and their associated 95% confidence intervals (CI), we used these data as summary statistics for each study. First, we derived the standard error of log (RR or OR) using the 95% CI, with the expression: [log (upper limit)−log (lower limit)]/2 × 1.96. These standard errors were used as weights for summary effect estimates in the meta-analysis. In quality score adjusted analysis, we combined quality score and the inverse of standard error for the weight based on the Shadish and Haddock formula, and used a modified ‘metagen’ function from the R-package ‘meta’ to implement the change (Shadish and Haddock, 1994). Given that many studies reported results separately for men and women, we included both estimates when reporting the overall association. In addition, we visually examined publication bias using Funnel plots and employed the rank correlation method to formally test for bias (Begg and Mazumdar, 1994).

## Results

We identified 507 potential studies. Of these we excluded those that were not conducted in humans (*n*=5), review studies (*n*=144), those whose outcome was not colon or colorectal cancer (*n*=163), editorials/letters to the editor/comments (*n*=8), those where physical activity was included only as a covariate and no data on the association with colon cancer was presented (*n*=22), those with prevalence data only (*n*=1), those where no physical activity data were presented (*n*=4), those that reported data published elsewhere (*n*=8), those with an English abstract but the body of the article was in a foreign language (*n*=1), leaving 60 studies. We then excluded studies that did not present data for colon cancer separately (as opposed to colorectal cancer) leaving 54 studies. When data from the same study was reported in more than one article (i.e., updated reports over time using the same baseline data), we included only the most recent publication. One exception was the Harvard Alumni Health Study where physical activity from two different adult time periods (i.e., different baselines) was used in the two publications (Lee *et al*, 1991; Lee and Paffenbarger, 1994). Finally, we also excluded those studies that did not report a relative risk and 95% confidence interval or crude data that allowed calculation of the relative risk and confidence interval (*n*=2). This left a total of 52 studies, 24 case–control (Vena *et al*, 1985; Brownson *et al*, 1989; Fredriksson *et al*, 1989; Gerhardsson de Verdier *et al*, 1990; Kato *et al*, 1990; Slattery *et al*, 1990, 1997; Whittemore *et al*, 1990; Vetter *et al*, 1992; Arbman *et al*, 1993; Vineis *et al*, 1993; Marcus *et al*, 1994; Kotake *et al*, 1995; White *et al*, 1996; La Vecchia *et al*, 1999; Levi *et al*, 1999; Tang *et al*, 1999; Tavani *et al*, 1999; Juarranz *et al*, 2002; Yeh *et al*, 2003; Fernandez *et al*, 2004; Hou *et al*, 2004; Isomura *et al*, 2006; Zhang *et al*, 2006) and 28 cohort studies (Garabrant *et al*, 1984; Gerhardsson *et al*, 1986, 1988; Lynge and Thygesen, 1988; Severson *et al*, 1989; Brownson *et al*, 1991; Lee *et al*, 1991, 1997, 2007; Dosemeci *et al*, 1993; Fraser and Pearce, 1993; Bostick *et al*, 1994; Lee and Paffenbarger, 1994; Thune and Lund, 1996; Colbert *et al*, 2001; Chao *et al*, 2004; Wei *et al*, 2004; Schnohr *et al*, 2005; Calton *et al*, 2006; Friedenreich *et al*, 2006; Johnsen *et al*, 2006; Larsson *et al*, 2006; Mai *et al*, 2007; Takahashi *et al*, 2007; Wolin *et al*, 2007; Howard *et al*, 2008; Moradi *et al*, 2008; Nilsen *et al*, 2008), in the present analyses. We found significant heterogeneity in the estimates across studies (*P*<0.0001) and thus employed random effects models. We found no statistical evidence of publication bias, using a funnel plot (*P* from rank correlation=0.45).

There was a significant 24% reduced risk of colon cancer when comparing the most *vs* least active individuals across all studies (RR=0.76, 95% CI: 0.72, 0.81). When we adjusted for quality score, the results held (RR=0.76, 95% CI: 0.74, 0.79).

**Figure 1:**
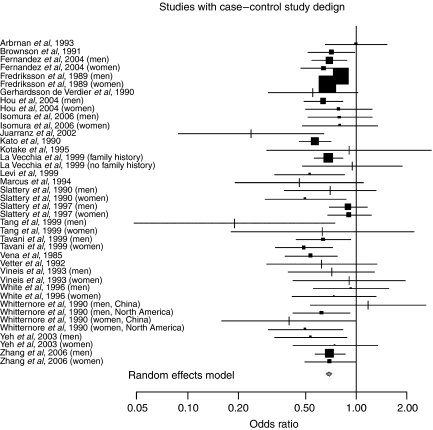
Meta-analysis of physical activity and colon cancer: case–control studies.

Examining case–control and cohort studies separately, we found significant risk reductions for both study designs, with the magnitude being larger for case–control (RR= 0.69, 95% CI: 0.65, 0.74), compared with cohort studies (RR=0.83, 95% CI: 0.78, 0.88) (Figure 1). When we examined men and women separately, we found similar results for both men (RR=0.76, 95% CI 0.71, 0.82) and women (RR=0.79, 95% CI: 0.71, 0.88). We further stratified the analyses by study design and gender, and observed generally similar results for men (RR=0.72, 95% CI: 0.66, 0.79) and women (RR=0.68, 95% CI: 0.64, 0.72) in case–control studies. For the cohort studies, the risk reduction appeared larger in men (RR=0.81, 95% CI: 0.73, 0.89), compared with women (RR=0.89, 95% CI: 0.81, 0.99).

When examining differences over time, we found that the inverse association was not different between studies published before 1993 (RR=0.74, 95% CI: 0.67, 0.82) and those published between 1993 and 1999 (RR=0.78, 95% CI: 0.70, 0.86) or after 1999 (RR=0.78, 95% CI: 0.73, 0.83).

Of the 24 case–control studies, 17 provided data separately on occupational physical activity and 10 provided separate data on leisure-time physical activity. For the 28 cohort studies, these numbers were 15 and 16, respectively. The findings from analyses that considered these domain-specific physical activities mirrored those from the overall analyses. Occupational physical activity was associated with a significant risk reduction (RR=0.78, 95% CI: 0.74, 0.83) for colon cancer. The effect was attenuated in cohort studies (RR=0.85, 95% CI: 0.77, 0.93) as compared to case–control studies (RR=0.73, 95% CI: 0.67, 0.79). Similarly, leisure-time physical activity was associated with a similar risk reduction over all studies (RR=0.77, 95% CI: 0.72, 0.82); the effect was attenuated in cohort (RR=0.82, 95% CI: 0.75, 0.87), compared with case–control (RR=0.69, 95% CI: 0.62, 0.78) studies.

## Discussion

Previous reviews of the association between physical activity and colon cancer have reported a risk reduction of approximately 30%, based on qualitative review, when comparing the most to the least active individuals (Lee and Oguma, 2006). Our formal meta-analysis of the data generally supports this, showing a 24% risk reduction overall, and generally similar risk reductions when men and women were examined separately. Several mechanisms have been proposed for the role of physical activity in reducing colon cancer risk including reduced insulin resistance and hyperinsulinemia, anti-inflammatory action, direct immune action, decreased intestinal transit time or higher vitamin D levels (Wolin *et al*, 2007). As future investigations explore these pathways, they will likely provide additional insights on the association between physical activity and colon cancer risk.

Although each study quantified activity differently limiting our ability to draw conclusions about the amount of physical activity necessary for the 24% risk reduction observed, a recent example provides some information. In the US Nurses' Health Study, Wolin *et al* (2007) report a 23% risk reduction when comparing the most to the least active women. The most active women expended more than 21.5 MET hours per week in leisure-time physical activity, whereas the least active expended less than 2 MET hours per week. These levels are equivalent to brisk walking for some 5–6 h per week in the most active and 0.5 h per week in the least active.

We found that the magnitude of risk reduction reported in case–control studies were stronger than those reported in cohort studies (30 *vs* 15% risk reduction), as also observed in a previous qualitative review (Lee and Oguma, 2006) and a previous meta-analysis (Samad *et al*, 2005). There are several possible reasons for this difference. Case–control studies may be subject to greater recall bias. Overall, we found no difference in risk reduction between men and women. This supports one previous qualitative review that found similar effects in women and men (Lee and Oguma, 2006), and contradicts suggestions that the beneficial effects of exercise may be attenuated in women (McTiernan *et al*, 2006; Mai *et al*, 2007). In their meta-analysis, Samad *et al* (2005) found a stronger association in men than women in cohort studies, but no difference in effects by gender in case–control studies . Similarly, we observed that cohort study results among women were less pronounced than those among men. This may be partly due to the small number of studies within each stratum. It may also reflect differences in the absolute physical activity values being compared, in that women typically report lower levels (intensity and duration) of physical activity than men, and higher levels of physical activity may be needed for risk reductions.

We found significant heterogeneity in the effects used in our analyses. This is not surprising given the variations in time of exposure assessment, length of follow-up, method of exposure assessment, type of physical activity assessed, levels of physical activity compared and covariates included in the analysis. To account for this heterogeneity, we used random effects models in our analyses.

**Figure 2:**
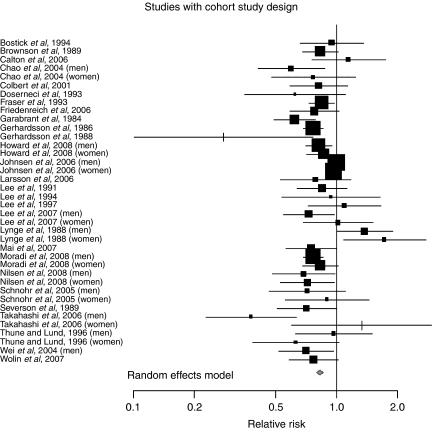
Meta-analysis of physical activity and colon cancer: cohort studies.

Most cohort studies formally evaluated the presence of a dose–response effect. Of the cohort studies, only four studies did not report a test of trend. Among the dose–response effects evaluated, less than half reported a significant trend. Of note, several studies that examined dose–response effects in men and women separately found significant trends only in men (Chao *et al*, 2004; Lee *et al*, 2007; Takahashi *et al*, 2007; Howard *et al*, 2008; Nilsen *et al*, 2008), but one found significant trends only in women (Thune and Lund, 1996) and two in both men and women (Wei *et al*, 2004; Moradi *et al*, 2008). In contrast, only 10 case–control studies reported a test for trend, and four found significant results for at least one group. Because the studies used different physical activity measures in their tests for trend (e.g., energy expended, intensity, frequency, or duration), as well as different categorisation schemes, we did not conduct a formal meta-analysis of trend across studies (Figure 2).

Increasing interest has focused on the type, intensity or duration of physical activity necessary for a protective effect (Physical Activity Guidelines Advisory Committee, 2008). We were able to examine the effect of physical activity domain (occupational *vs* leisure-time) and found that the results were similar. Few studies have reported results in sufficient detail to allow a formal evaluation of the different effects of intensity or duration. Qualitative evaluations have suggested that vigorous physical activity may be necessary to reduce the risk (Slattery, 2004) though others have concluded that sufficient durations of moderate or vigorous intensity physical activity are likely to reduce the risk of colon cancer (Lee and Oguma, 2006). Recently, it has been suggested that walking alone may be sufficient to reduce risk (Takahashi *et al*, 2007; Wolin *et al*, 2007) though not all studies agree (Chao *et al*, 2004). Sufficient number of studies have not reported on the benefits of walking to allow formal evaluation of the effect. As additional studies report on the separate effects of intensity, duration and physical activity type, analyses of this data should be undertaken as these details are important to inform public health recommendations. Additional studies may also examine modification of the effect by race/ethnicity, BMI, diet and tumour location as the quantity of data on those factors increases.

We have previously hypothesised (Wolin *et al*, 2007) that the association between physical activity and colon cancer may be attenuating over time as screening decreases the number of colon tumours overall and distal colon tumours in particular. Fraser and Pearce (1993) examined secular trends in the association between occupational physical activity and risk of colon cancer and found that the association was stronger in the earlier era examined. However, this should be interpreted cautiously as other factors, including changes in the quality of physical activity assessment, may also contribute. In addition, the later studies also tended to adjust for larger numbers of potential confounders, typically attenuating relative risks. We found little evidence for a difference over time when we stratified the meta-analysis by publication year.

In conclusion, this meta-analysis provides additional support for the inverse association between physical activity and colon cancer. It provides a formal estimate showing that individuals can likely reduce their risk of colon cancer, overall, by 24% through participation in physical activity. Additional research on the type, intensity, and duration of physical activity that may afford the greatest risk reduction will inform public health recommendations regarding quantification of specific physical activity details.
